# Unidirectional drying of a suspension of diffusiophoretic colloids under gravity

**DOI:** 10.1039/d3ra00115f

**Published:** 2023-03-20

**Authors:** Jinjie Xu, Zhikui Wang, Henry C. W. Chu

**Affiliations:** a Department of Chemical Engineering, University of Florida Gainesville FL 32611 USA h.chu@ufl.edu

## Abstract

Recent experiments (K. Inoue and S. Inasawa, *RSC Adv.*, 2020, **10**, 15763–15768) and simulations (J.-B. Salmon and F. Doumenc, *Phys. Rev. Fluids*, 2020, **5**, 024201) demonstrated the significant impact of gravity on unidirectional drying of a colloidal suspension. However, under gravity, the role of colloid transport induced by an electrolyte concentration gradient, a mechanism known as diffusiophoresis, is unexplored to date. In this work, we employ direct numerical simulations and develop a macrotransport theory to analyze the advective–diffusive transport of an electrolyte-colloid suspension in a unidirectional drying cell under the influence of gravity and diffusiophoresis. We report three key findings. First, drying a suspension of solute-attracted diffusiophoretic colloids causes the strongest phase separation and generates the thinnest colloidal layer compared to non-diffusiophoretic or solute-repelled colloids. Second, when colloids are strongly solute-repelled, diffusiophoresis prevents the formation of colloid concentration gradient and hence gravity has a negligible effect on colloidal layer formation. Third, our macrotransport theory predicts new scalings for the growth of the colloidal layer. The scalings match with direct numerical simulations and indicate that the colloidal layer produced by solute-repelled diffusiophoretic colloids could be an order of magnitude thicker compared to non-diffusiophoretic or solute-attracted colloids. Our results enable tailoring the separation of colloid-electrolyte suspensions by tuning the interactions between the solvent, electrolyte, and colloids under Earth's or microgravity, which is central to ground-based and in-space applications.

## Abstract 2

We conduct numerical simulations and develop a macrotransport theory to show a diffusiophoresis-based, order-of-magnitude enhancement in the thickness of the colloidal layer produced by unidirectional drying of an electrolyte-colloid suspension.

## Graphical abstract

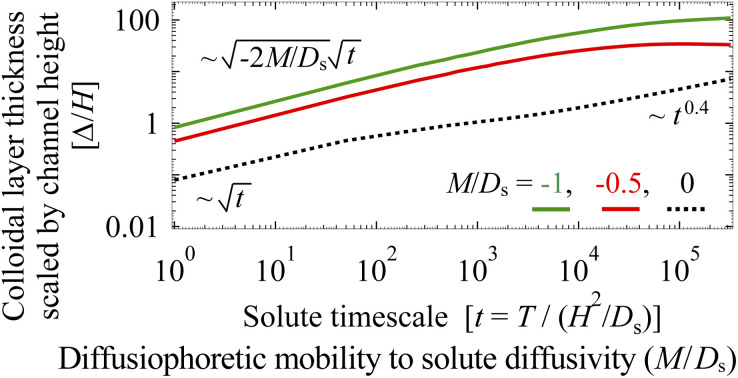

## 1 Introduction

Unidirectional drying of a colloidal suspension has been used widely for manufacturing microstructured materials, such as ceramics, electrodes, and photonic crystals.^1–7^ A typical experimental setup of unidirectional drying involves depositing a mixture of colloids and a volatile solvent into a microchannel.^8–20^ One end of the channel is connected to a large reservoir which provides a constant supply of the mixture to the channel. Evaporation occurs at the other end of the channel, the drying interface, which opens to the atmosphere. Solvent evaporation induces a flow of the mixture toward the drying interface. The colloids are carried by the solvent and concentrate at the drying interface, forming a colloidal film. Recent experiments^19^ and simulations^21^ demonstrated that gravity plays an important role in the phase separation process. Specifically, under evaporation of a non-electrolyte-colloid suspension, colloid concentration increases on approaching the drying interface. Sedimentation of colloids causes a backflow of the mixture away from the drying interface, which enables a continuous growth of the colloidal film.

Here, we hypothesize that phase separating an electrolyte-colloid suspension could be drastically different from that of a non-electrolyte-colloid suspension due to a mechanism known as diffusiophoresis.^22–26^ Diffusiophoresis refers to the deterministic motion of particles induced by a surrounding concentration gradient of solute. Diffusiophoresis has received much attention in recent years for its ability to manipulate colloid transport in a wide range of applications, including mixing and separation,^27–49^ enhanced oil recovery,^50–52^ and drug delivery.^53,54^ In our hypothesis, we envision that evaporation will induce an electrolyte concentration gradient, by the same token as that of the colloid, where the electrolyte concentration will increase toward the drying interface. The electrolyte gradient will in turn induce diffusiophoretic motion of colloids, which will drastically alter the colloid transport. The diffusiophoretic velocity of a colloid is given by ***V*** = *M*∇log *S*,^22–26^ where *S* is the ionic solute concentration and the mobility *M* encompasses information of the electrolyte and colloid such as the ion valence and colloid surface potential. The mobility can be positive or negative, corresponding to diffusiophoresis driving colloids up (solute-attracted) or down (solute-repelled) the solute gradient, respectively. The diffusiophoretic velocity (∼10^−6^ m s^−1^)^25,34^ is typically comparable to or orders of magnitude larger than the evaporation-induced fluid flow that carries the colloids in a drying cell (∼10^−9^ to 10^−6^ m s^−1^).^4,7,55^ This strengthens our hypothesis that the phase separation of an electrolyte-colloid suspension could be drastically different from that of a non-electrolyte-colloid suspension.

In this work, we utilize direct numerical simulations and develop a macrotransport theory to analyze the advective–diffusive transport of an electrolyte-colloid suspension in a unidirectional drying cell. The electrolyte and colloid motion are influenced by diffusiophoresis, gravity, and solvent evaporation. We report three key findings that confirm our hypothesis. First, there is a strong phase separation in drying a suspension of solute-attracted colloids, which generates the thinnest colloidal layer relative to drying a suspension of non-diffusiophoretic or solute-repelled colloids. Second, when colloids are solute-attracted or weakly solute-repelled, gravity could affect the colloid transport and thickness of the colloidal layer substantially. However, when colloids are strongly solute-repelled, diffusiophoresis could nullify the effect of gravity on colloid transport by eliminating the formation of a significant colloid concentration gradient. Third, our macrotransport theory predicts new early-time and long-time scalings of the growth of the colloidal layer which agree with direct numerical simulations. The colloidal layer generated by solute-repelled colloids could be ten times thicker than that by non-diffusiophoretic colloids.

The rest of this article is outlined as follows. In Section 2, we formulate the problem by presenting the governing equations and boundary conditions for the transport of the solvent, ionic solute, and colloids. Derivations of the macrotransport theory of diffusiophoretic colloid transport under varying strengths of gravity as well as scalings of the growth of the colloidal layer are presented in Appendix A. In Section 3, we present our results and elaborate on the three above-mentioned key findings. In Section 4, we summarize this study and offer ideas for future work.

## 2 Problem formulation

**Fig. 1:**
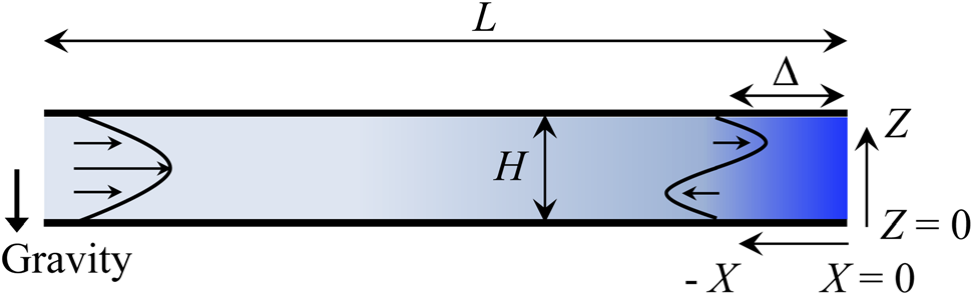
Unidirectional drying of an electrolyte-colloid suspension in a channel that consists of two parallel plates of length *L* separated by a distance *H*. The left-end of the channel is connected to a large reservoir which provides a constant supply of the suspension to the channel. Evaporation induces a flow of the suspension with a constant velocity at the drying interface, the right-end of the channel. A colloidal layer of thickness *Δ* is formed at the drying interface.

Consider a channel that consists of two parallel plates of length *L* separated by a distance *H* (Fig. 1). Initially, the channel is filled uniformly with a dilute suspension of constant density, *ρ*_i_, comprising a volatile solvent of kinematic viscosity *ν*_s_, a non-volatile ionic solute of concentration *S*_i_, and non-volatile colloids of concentration *C*_i_. The left-end of the channel is connected to a large reservoir of the suspension. Evaporation induces a flow of the suspension with a constant velocity at the drying interface, the right-end of the channel. A colloidal layer of thickness *Δ* is formed at the drying interface.

When colloids and solute concentrate on approaching the drying interface, the density of the mixture increases. The local density of the mixture, *ρ*, is related to the local concentration of the colloid, *C*, and solute, *S*, *via*^21,56^

1 *ρ* = *ρ*_i_[1 + *β*_c_(*C* − *C*_i_) + *β*_s_(*S* − *S*_i_)],

where *β*_c_ and *β*_s_ are the solutal expansion coefficient of the colloid and the solute, respectively. A difference in the density induces a gravitational body force. Under the Boussinesq approximation for microscale flows,^21,56–59^ this gravitational force appears in the Stokes equation that governs the fluid motion, along with the continuity equation

2 *ρ*_i_*ν*_s_∇^2^***U*** − ∇*P* + (*ρ* − *ρ*_i_)***g*** = **0** and ∇·***U*** = 0,

where ***U*** is the solvent flow velocity, *P* is the pressure deviation from the initial hydrostatic pressure, and ***g*** is the gravitational acceleration. The evolution of the solute concentration is governed by the advection–diffusion equation3
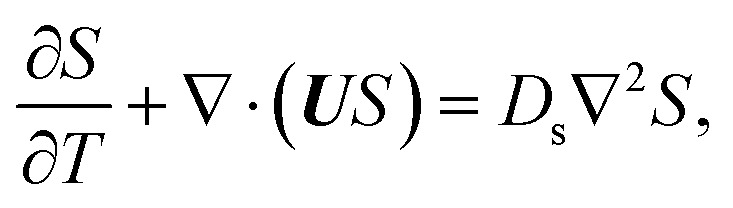
where *T* is time and *D*_s_ is the solute diffusivity. A solute concentration gradient is developed over time, which induces a diffusiophoretic velocity of the colloid, ***V*** = *M*∇log *S*.^22–26^ The evolution of the colloid concentration is governed by the advection–diffusion equation which comprises the diffusiophoretic velocity^39–41^4
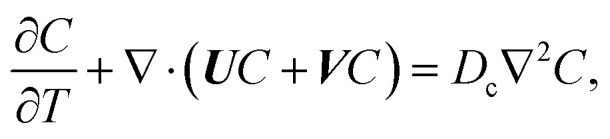
where *D*_c_ is the colloid diffusivity. Following prior work, the colloidal layer thickness, *Δ*, and the mean position of the colloid distribution, *Ω*, are defined as^21,42^5
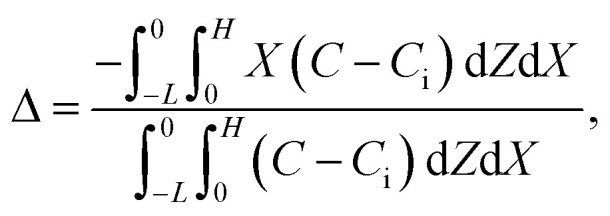
and6
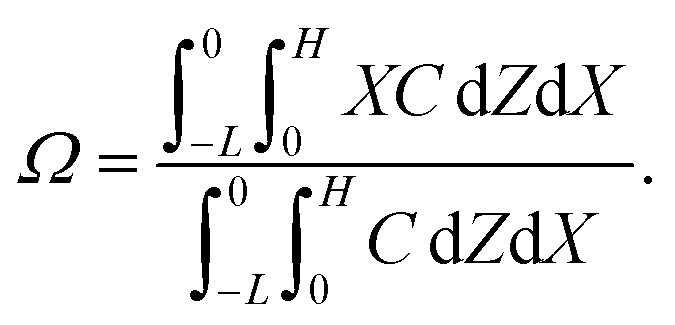


The initial and boundary conditions that accompany eqn (1)–(4) are as follows. The initial conditions at *T* = 0 are

7 *C* = *C*_i_ and *S* = *S*_i_.

For the boundary conditions, at the left-end of the channel, *X* = −*L*, connection to a large reservoir of suspension requires that

8 *C* = *C*_i_ and *S* = *S*_i_.

At the channel walls, *Z* = 0 and *Z* = *H*, no hydrodynamic slip and no penetration of the solvent require that

9 ***U*** = **0**.

Diffusioosmosis adjacent to the channel walls is ignored in the present study to highlight the effect of diffusiophoresis. In practice, diffusioosmosis can be mitigated by precoating the channel walls with a mono-molecular layer of non-cross-linked polyacrylamide.^60,61^ No penetration of the colloids and solute requires that10
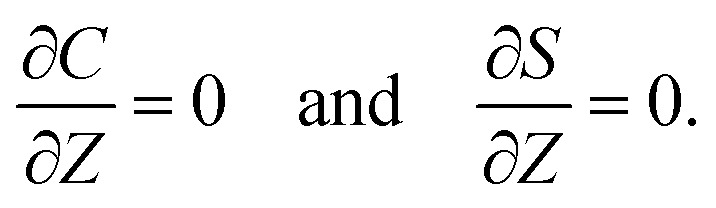
At the drying interface, *X* = 0, it requires that

11 *U*_*X*_ = *E*,

12
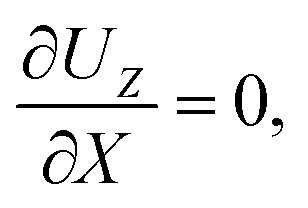
13
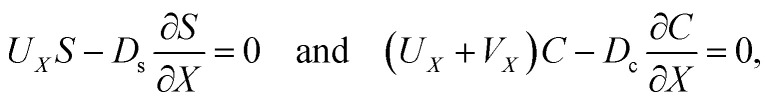
where eqn (11) represents that the solvent velocity in the *X*-direction, *U*_*X*_, equals the evaporation rate *E*,^21^eqn (12) represents that the drying interface is a free surface, and eqn (13) ensures the non-volatility of the solute and colloids.

We introduce the following non-dimensionalization scheme,14
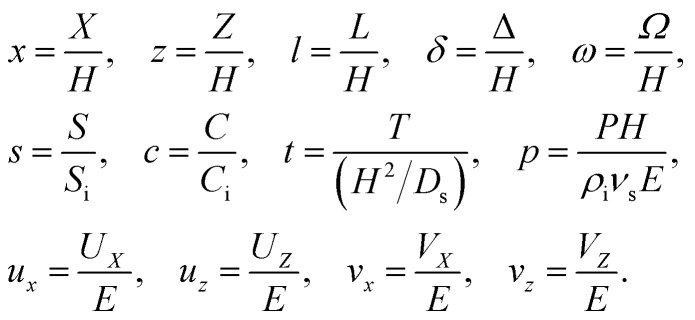
Upon non-dimensionalization, eqn (1)–(4) become15
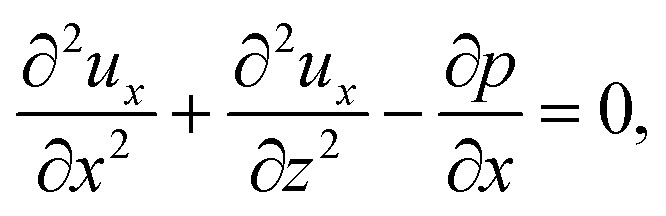
16
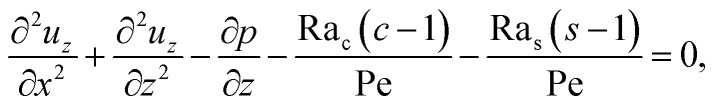
17
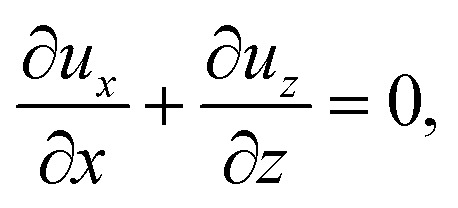
18
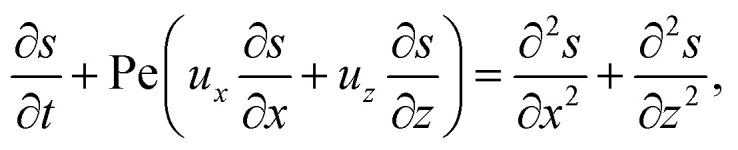
19
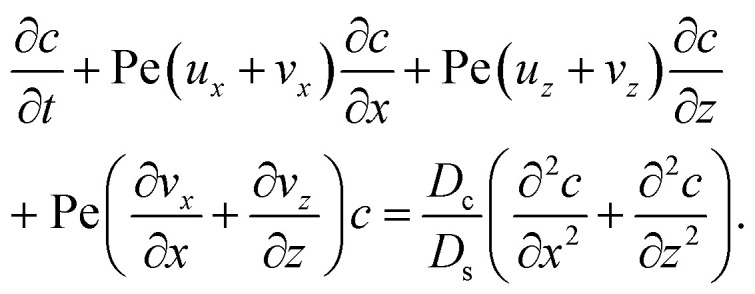
Five dimensionless groups emerge. These include two Rayleigh numbers of the colloids and the solute, Ra_c_ = *β*_c_*gH*^3^*C*_i_/(*ν*_s_*D*_s_) and Ra_s_ = *β*_s_*gH*^3^*S*_i_/(*ν*_s_*D*_s_), which describe the relative strength between gravity and diffusion; a Peclet number, Pe = *EH*/*D*_s_, which describes the relative strength between solvent convection and solute diffusion; the ratio of the colloid to solute diffusivity, *D*_c_/*D*_s_; and the ratio of the diffusiophoretic mobility to solute diffusivity, *M*/*D*_s_. The non-dimensionalized initial conditions are *c* = 1 and *s* = 1 at *t* = 0. The non-dimensionalized boundary conditions are as follows. At the left-end of the channel, *x* = −*l*, *c* = 1 and *s* = 1. At the channel walls, *z* = 0 and *z* = 1, ***u*** = **0**, ∂*c*/∂*z* = 0, and ∂*s*/∂*z* = 0. At the drying interface, *x* = 0, *u*_*x*_ = 1, ∂*u*_*z*_/∂*x* = 0, Pe*u*_*x*_*s* − ∂*s*/∂*x* = 0, and Pe(*u*_*x*_ + *v*_*x*_)*c* − (*D*_c_/*D*_s_)∂*c*/∂*x* = 0.

The physical range of the five dimensionless groups can be obtained from the dimensional parameters. Namely, *H* ∈ [10^−6^, 10^−4^] m, *E* ∈ [10^−9^, 10^−6^] m s^−1^,^4,7,55^*D*_s_ ∼ 10^−9^ m^2^ s^−1^, *D*_c_ ∈ [10^−13^, 10^−11^] m^2^ s^−1^,^34^*M* ∈ [−10^−9^, 10^−9^] m^2^ s^−1^,^34,42,62^*ν*_s_ ∼ 10^−6^ m^2^ s^−1^, *g* ∈ [0, 9.8] m s^−2^, *C*_i_ ∈ [0, 10^−4^], *β*_c_ ∼ 1, *S*_i_ ∈ [0, 0.1] mol m^−3^,^34,38^ and *β*_s_ ∼ 10^−5^ m^3^ mol^−1^.^21,63–66^ Here, we choose *β*_s_ = 4 × 10^−5^ m^3^ mol^−1^ that corresponds to a sodium chloride solution and *β*_c_ ≈ *ρ*_c_/*ρ*_s_ − 1 = 1.2 that is based on the density of silica (colloid) *ρ*_c_ = 2200 kg m^−3^ and water (solvent) *ρ*_s_ = 1000 kg m^−3^. Hence, the ranges of the five dimensionless groups are Ra_c_ ∈ [0, 1.2], Ra_s_ ∈ [0, 4 × 10^−2^], Pe ∈ [10^−6^, 10^−1^], *D*_c_/*D*_s_ ∈ [10^−4^, 10^−2^], and *M*/*D*_s_ ∈ [−1, 1]. With these parameters, we solve the non-dimensionalized eqn (15)–(19) using the ‘Creeping flow’ and ‘Stabilized Convection–Diffusion Equation’ modules in COMSOL Multiphysics. The implicit ‘backward differentiation formula (BDF) solver’ and ‘adaptive time stepping’ are selected to capture the solute and fluid transport on the fast, solute diffusive time scale, *H*^2^/*D*_s_. Spatial discretization is achieved by a structured mesh of free triangular elements. The convergence of solution has been tested by successive mesh refinements.

## 3 Results and discussion

In this section, we examine the time evolution of the *x*-component solvent velocity *u*_x_, solute concentration *s*, *x*-component diffusiophoretic velocity *v*_x_, colloid concentration *c*, colloidal layer thickness *δ*, and the mean position of the colloid distribution *ω*. We have conducted all simulations with a channel of length much larger than the channel height, *l* = 10^3^, so that key flow features developed near the drying interface, *e.g.*, fluid backflow, are not hindered by the presence of the mixture reservoir. In all simulations, the maximum colloid volume fraction *C* < 0.05 (maximum *c* < 500) so that particle–particle interactions are negligible.^34^ We start by showing the impact of varying strengths of diffusiophoresis (*M*/*D*_s_) in Section 3.1. This is followed by showing the impact of varying strengths of gravity (Ra_c_ and Ra_s_) in Section 3.2.

### 3.1 Impact of varying strengths of diffusiophoresis

#### 3.1.1 Velocity and concentration fields

**Fig. 2:**
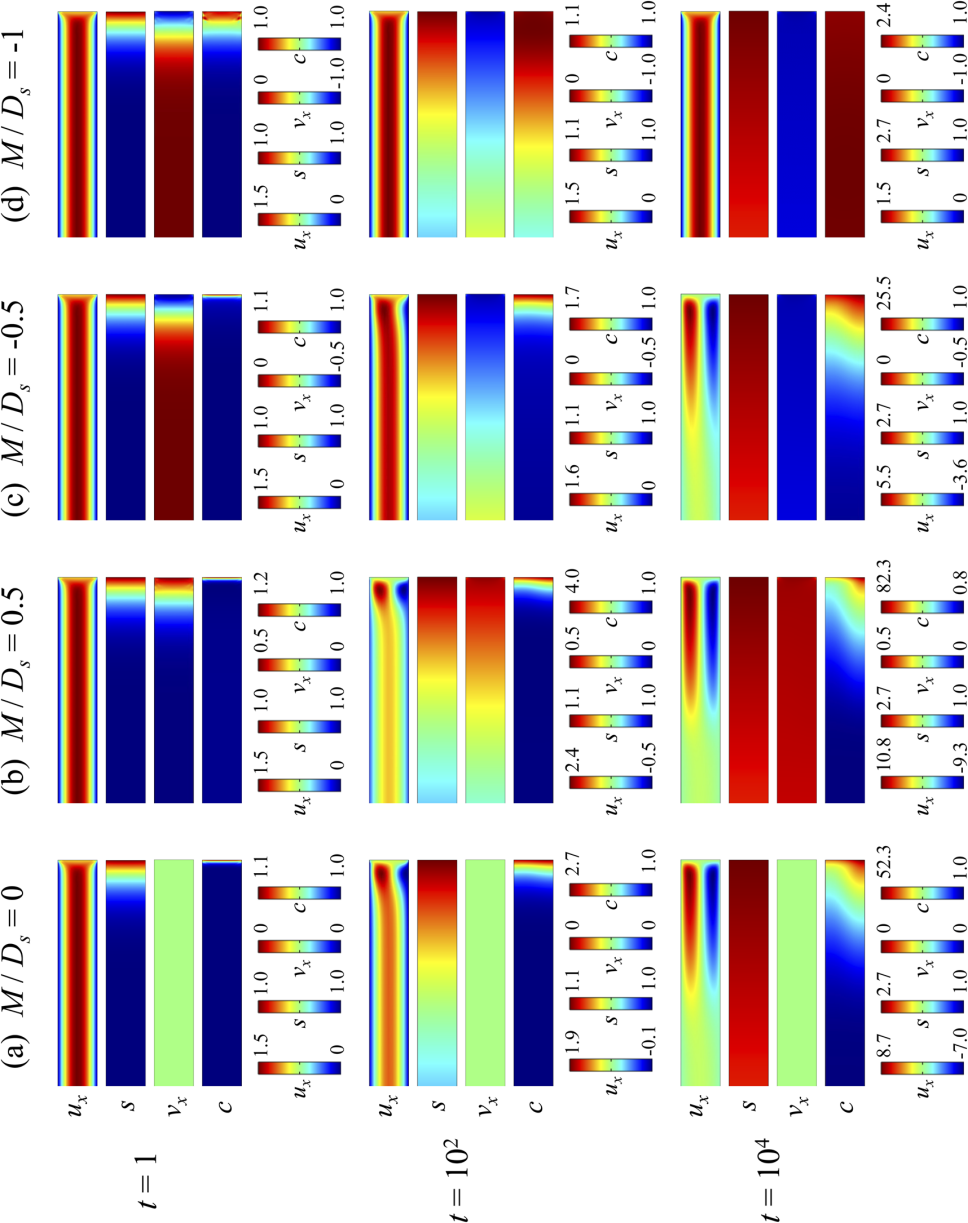
Density profiles of the *x*-component fluid velocity, *u*_x_, solute concentration, *s*, *x*-component diffusiophoretic velocity, *v*_x_, and colloid concentration, *c*, at different times, *t*, with *D*_c_/*D*_s_ = 10^−2^, Pe = 10^−2^, Ra_c_ = 1.2, and Ra_s_ = 4 × 10^−2^. The density profiles are obtained with channel length *l* = 10^3^ but only the sections next to the drying interface, *x* ∈ [−10, 0], are shown to illustrate important physics. (a) *M*/*D*_s_ = 0; no diffusiophoresis. (b) *M*/*D*_s_ = 0.5; solute-attracted diffusiophoresis. (c) *M*/*D*_s_ = −0.5; weakly solute-repelled diffusiophoresis. (d) *M*/*D*_s_ = −1; strongly solute-repelled diffusiophoresis.

Fig. 2 shows twelve sets of density profiles of the *x*-component solvent velocity, *u*_x_, solute concentration, *s*, *x*-component diffusiophoretic velocity, *v*_x_, and colloid concentration, *c* at different times *t* and *M*/*D*_s_ with *D*_c_/*D*_s_ = 10^−2^, Pe = 10^−2^, Ra_c_ = 1.2, and Ra_s_ = 4 × 10^−2^. The top and bottom of each density plot corresponds to the channel walls at *z* = 1 and *z* = 0, respectively. The right-end of each density plot corresponds to the drying interface at *x* = 0. To illustrate important physics, we show only the section of the channel next to the drying interface, *x* ∈ [−10, 0], instead of the entire channel, *x* ∈ [−10^3^, 0]. The vertical bars below each set of density profiles show the values of the density profiles.

Let us first examine Fig. 2(a) which is obtained with *M*/*D*_s_ = 0 and corresponds to no diffusiophoresis in the system. This recovers the key observations in prior work^21^ and validates our simulation framework. We state and explain the observations as follows. At early times, *t* = 1, evaporation induces a net solvent flow to the right across any cross section of the channel, as prescribed by the boundary condition at the drying interface. Indeed, shown in the second and fourth panel, the solvent flow carries the solute and colloids to the right and they are accumulating near the drying interface. However, the colloid (∂*c*/∂*x*) and solute (∂*s*/∂*x*) concentration gradients are not significant near the drying interface and hence there is no fluid backflow. This can be understood by the scaling of the solvent backflow velocity,20
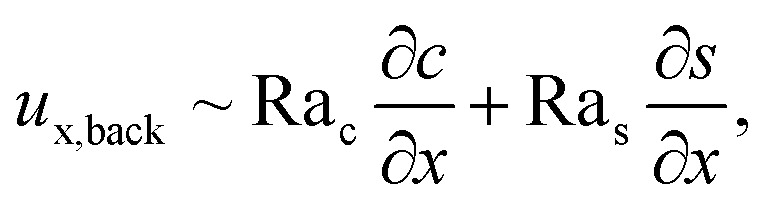
which is obtained from a balance between the viscous and buoyancy terms in eqn (15) and (16).^21,67^ That is, *u*_x,back_ → 0 as ∂*c*/∂*x* → 0 and ∂*s*/∂*x* → 0. As a result, the parabolic flow profile resembles a pressure-driven flow with the maximum velocity at the centerline of the channel (*z* = 1/2) and zero velocity at the channel walls (*z* = 0 and *z* = 1) due to no hydrodynamic slip.

Going from *t* = 1 to 10^2^ in Fig. 2(a), solvent flow continues to carry colloids and solute towards the drying interface. The colloid and solute concentration gradients near the drying interface strengthen. Hence, the parabolic flow profile weakens at *t* = 10^2^. Instead, colloids undergo sedimentation and induce a backflow of the suspension as shown in the first panel where, in the bottom half of the channel, the suspension flows to the left as indicated by a negative *u*_x_. As time goes by at *t* = 10^4^, the colloid and solute concentration gradients near the drying interface lengthen in the *x*-direction and continue to strengthen. As a result, the solvent flow profile develops fully, with a flow toward and away from the drying interface in the upper-half and lower-half of the channel, respectively. The backflow increases in magnitude according to eqn (20). Note that, during evolution, the colloid concentration profile, *c*, is increasingly asymmetric about the centerline of the channel, which follows from the asymmetry of the solvent flow.

As an overview of Fig. 2(a), the diffusiophoretic velocity, *v*_x_, is zero everywhere at all times, confirming that the solute gradient induces no diffusiophoresis to colloids due to the present case of *M*/*D*_s_ = 0 (recall that ***V*** = *M*∇log *S*). On a different note, distinct from the asymmetric colloid distribution, *c*, about the channel centerline due to the solvent backflow, the solute distribution, *s*, is symmetric. This can be understood by examining the relation *H*^2^/*D*_s_ = Pe(*H*/*E*) with Pe = 10^−2^ in the present case. Physically, the solute takes a much shorter time to diffuse across the channel height (*H*^2^/*D*_s_) compared to it being transported by the backflow (*H*/*E*) for a unit distance in the *x*-direction (remember that *x* = *X*/*H*). In other words, diffusion has made uniform the solute distribution across the channel height before the distribution gets distorted by the backflow in the *x*-direction. Hence, the solute concentration is uniform in the *z*-direction at any position *x*.

Next, let us look at Fig. 2(b) that is obtained with *M*/*D*_s_ = 0.5 and corresponds to solute-attracted diffusiophoresis in the system. The presence of solute-attracted diffusiophoresis is confirmed by a positive diffusiophoretic velocity, *v*_x_ (up the solute gradient to the right), at all times. At an early time, *t* = 1, comparing Fig. 2(b) and (a), the solvent flow velocity *u*_x_ in panel (b) and (a) are identical. Physically, this means that diffusiophoresis does not alter the solvent flow so long as solvent backflow is absent. Furthermore, the colloid concentration, *c*, and concentration gradient, ∂*c*/∂*x*, in Fig. 2(b) are higher than those in Fig. 2(a). This is because, under solute-attracted diffusiophoresis, there is an additional diffusiophoretic velocity of the colloids whose direction is up the solute gradient to the right, transporting more colloids toward the drying interface compared to no diffusiophoresis.

At long times, *t* = 10^2^ and *t* = 10^4^, comparing Fig. 2(b) and (a), the magnitude of *u*_x_ and *c* in panel (b) are higher than those in panel (a) at the same time *t*, although the profiles of *u*_x_, *s*, and *c* between panel (b) and (a) at the same *t* are qualitatively the same. These observations can be understood as follows. As noted above, solute-attracted diffusiophoresis increases *c* and ∂*c*/∂*x* near the drying interface. The solvent (backflow) velocity also increases according to eqn (20). On a different note, a colloidal layer of high colloid concentration is formed near the drying interface and is thinner than that in Fig. 2(a) with no diffusiophoresis, meaning that solute-attracted diffusiophoresis causes strong phase separation.

Next, let us examine Fig. 2(c) that is obtained with *M*/*D*_s_ = −0.5 and corresponds to weakly solute-repelled diffusiophoresis. The phenomena demonstrated in Fig. 2(c) are the opposite of Fig. 2(b). First, the presence of solute-repelled diffusiophoresis is confirmed by a negative diffusiophoretic velocity, *v*_x_ (down the solute gradient to the left), at all times. At long times, *t* = 10^2^ and *t* = 10^4^, comparing Fig. 2(c) and (a), the magnitude of *u*_x_ and *c* in panel (c) are lower than those in panel (a) at the same time *t*, although the profiles of *u*_x_, *s*, and *c* between panel (c) and (a) at the same *t* are qualitatively the same. These observations can be understood as follows. Solute-repelled diffusiophoresis induces a convective flux of colloids down the solute gradient to the left, which partially cancels the convective flux of colloids up the solute gradient to the right due to the evaporation-induced solvent flow. This leads to an overall weaker transport of colloids toward the drying interface in Fig. 2(c) compared to Fig. 2(a). As a result, the colloid concentration and concentration gradient decrease, and hence the backflow velocity decreases according to eqn (20). Also, note that the colloidal layer formed near the drying interface is thicker than that in Fig. 2(a), meaning that solute-repelled diffusiophoresis weakens phase separation.

Next, let us look at Fig. 2(d) that is obtained with *M*/*D*_s_ = −1 and corresponds to strongly solute-repelled diffusiophoresis. The presence of strongly solute-repelled diffusiophoresis is confirmed by a more negative diffusiophoretic velocity, *v*_x_, relative to Fig. 2(c). Notably, at *t* ≥ 10^2^, the solvent flow profile, *u*_x_, and colloid distribution, *c*, are qualitatively different than all previous cases in Fig. 2(a)–(c). Specifically, solvent backflow no longer exists. This is because the diffusiophoretic flux of colloids to the left is strong enough to counter a significant portion of that due to solvent convection to the right. As a result, the colloid concentration is nearly uniform, where the maximum and minimum values of *c* are 2.4 and 1, respectively, even at *t* = 10^4^. Therefore, the colloid concentration gradient built up is too weak to generate a solvent backflow. Phase separation is the weakest in this case, resulting in the thickest colloidal layer. In the next section, we quantify the time evolution of the colloidal layer thickness and mean position of the colloid distribution in the above cases.

#### 3.1.2 Colloidal layer thickness and mean colloid position

**Fig. 3:**
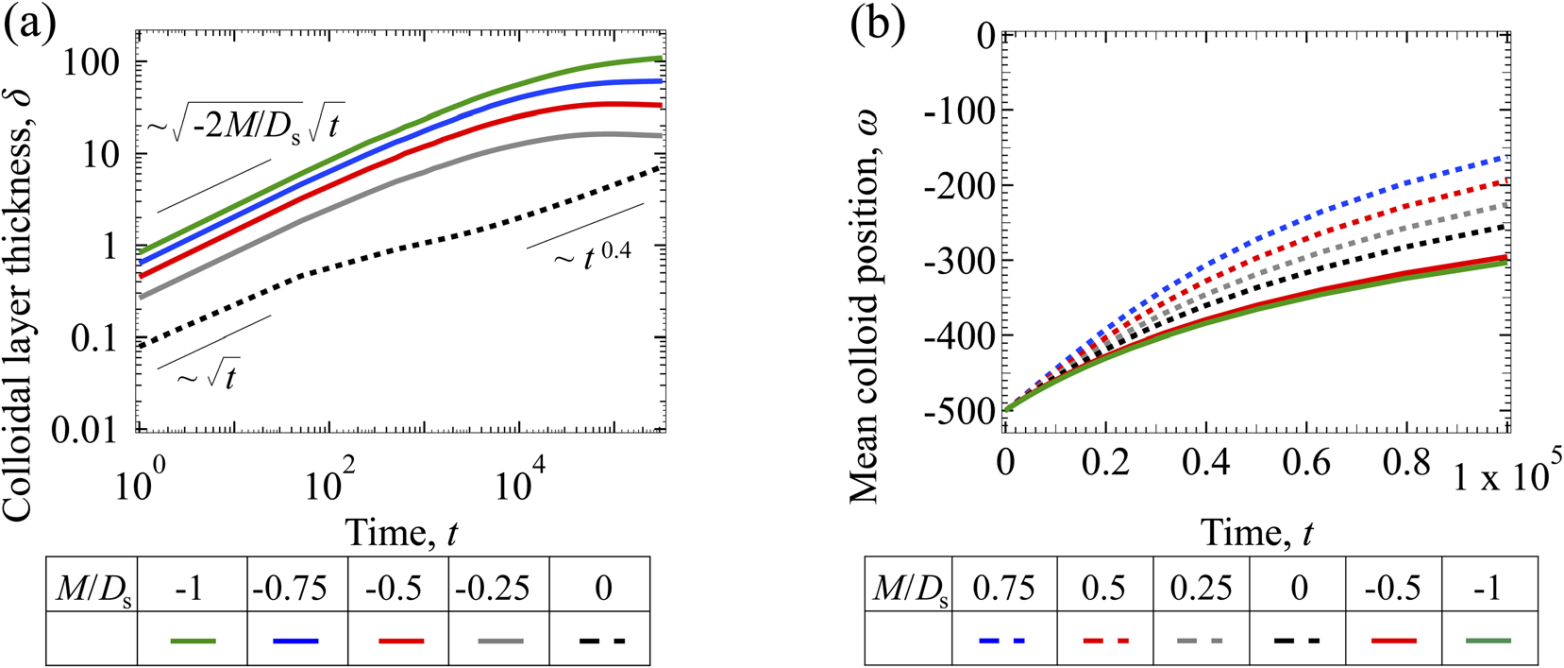
Time evolution of the (a) colloidal layer thickness, *δ*, and (b) mean position of the colloid distribution, *ω*, for different *M*/*D*_s_ with *D*_c_/*D*_s_ = 10^−2^, Pe = 10^−2^, Ra_c_ = 1.2, and Ra_s_ = 4 × 10^−2^.

Fig. 3(a) shows the time evolution of the colloidal layer thickness, *δ*, for different non-positive *M*/*D*_s_, with *D*_c_/*D*_s_ = 10^−2^, Pe = 10^−2^, Ra_c_ = 1.2, and Ra_s_ = 4 × 10^−2^. In the following, we analyze the results and highlight the scalings of the growth of the colloidal layer thickness. We remark that the scalings obtained from direct numerical simulations in Fig. 3(a) agree with those obtained from a macrotransport theory. Readers are referred to Appendix A for detailed derivations of the macrotransport theory.

Let us start by analyzing the case with no diffusiophoresis (dashed line) in Fig. 3(a). The colloidal layer thickness grows diffusively as 
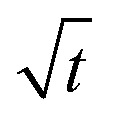
 at early times and grows as *t*^0.4^ at long times due to a balance of convection and gravity [eqn (37) and (39)]. This recovers the results of prior work.^21^

Next, let us analyze the cases with solute-repelled diffusiophoresis, shown by solid lines in Fig. 3(a). Regardless of the strengths of diffusiophoresis, *M*/*D*_s_, our simulations show a new early-time scaling, where *δ* grows as 
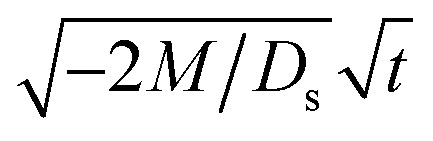
. Our macrotransport theory recovers this scaling and identifies that this scaling is due to a balance between transient and diffusiophoretic transport of colloids [eqn (33)]. The prefactor of the scaling, 
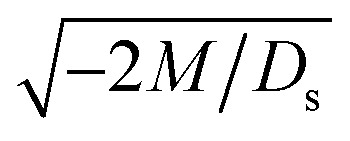
, also correctly predicts the trend shown in Fig. 3(a) where, at a fixed *t*, *δ* increases as *M*/*D*_s_ becomes more negative.

**Fig. 4:**
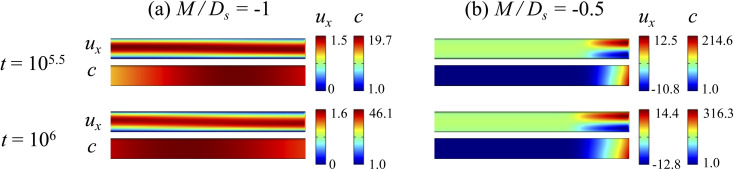
Density profiles of the *x*-component fluid velocity, *u*_x_, and colloid concentration, *c*, at different times, *t*, with *D*_c_/*D*_s_ = 10^−2^, Pe = 10^−2^, Ra_c_ = 1.2, and Ra_s_ = 4 × 10^−2^. The density profiles are obtained with channel length *l* = 10^3^ but only the sections next to the drying interface, *x* ∈ [−10^2^, 0], are shown to illustrate important physics. (a) *M*/*D*_s_ = −1; strongly solute-repelled diffusiophoresis. (b) *M*/*D*_s_ = −0.5; weakly solute-repelled diffusiophoresis.

Diffusiophoresis also alters the long-time scaling of *δ*. In Fig. 3(a), our simulations show that the scaling transition from *t*^0.4^ at *M*/*D*_s_ = 0 to a plateau at *M*/*D*_s_ = −0.25. Our macrotransport theory recovers this plateau and shows that it is due to a balance between fluid convection and diffusiophoresis [eqn (34)]. The plateau persists up to *M*/*D*_s_ = −0.75. However, for strong diffusiophoresis where *M*/*D*_s_ = −1, *δ* grows continuously and deviates from the plateau. We identify that this deviation is due to a phenomenon that the peak of the colloid distribution is transported away from the drying interface, as shown in Fig. 4(a). This phenomenon is unique for systems with strongly solute-repelled diffusiophoresis, which is in contrast to systems with weakly solute-repelled diffusiophoresis (and solute-attracted diffusiophoresis or no diffusiophoresis) where the peak of the colloid distribution stays at the drying interface at all times, as shown in Fig. 4(b). As an overview of Fig. 3(a), the colloidal layer generated by solute-repelled diffusiophoretic colloids could be an order of magnitude thicker than that by non-diffusiophoretic colloids, highlighting the impact of diffusiophoresis on the production of colloidal films.

For the cases with solute-attracted diffusiophoresis, *δ* is not applicable to quantify the colloid distribution because the colloid concentration *C* at some positions are smaller than the initial colloid concentration *C*_i_, leading to a negative *δ* which is physically irrelevant [eqn (5)]. To still quantify the colloid distribution, in Fig. 3(b) we show the mean position of the colloid distribution, *ω*, for different *M*/*D*_s_. Note that *ω* is also applicable to quantify cases with solute-repelled diffusiophoresis and we show their data in Fig. 3(b) for comparison. Fig. 3(b) shows that, at a fixed *t*, *ω* approaches *x* = 0 monotonically as *M*/*D*_s_ becomes more positive. Physically, as *M*/*D*_s_ increases from negative to positive, the colloid diffusiophoretic velocity *v*_x_ switches from directed-away to directed-toward the drying interface where the solute accumulates, transporting more colloids toward the drying interface. Thus, the mean position of the colloid distribution shifts toward the drying interface at *x* = 0.

### 3.2 Impact of varying strengths of gravity

#### 3.2.1 Velocity and concentration fields

**Fig. 5:**
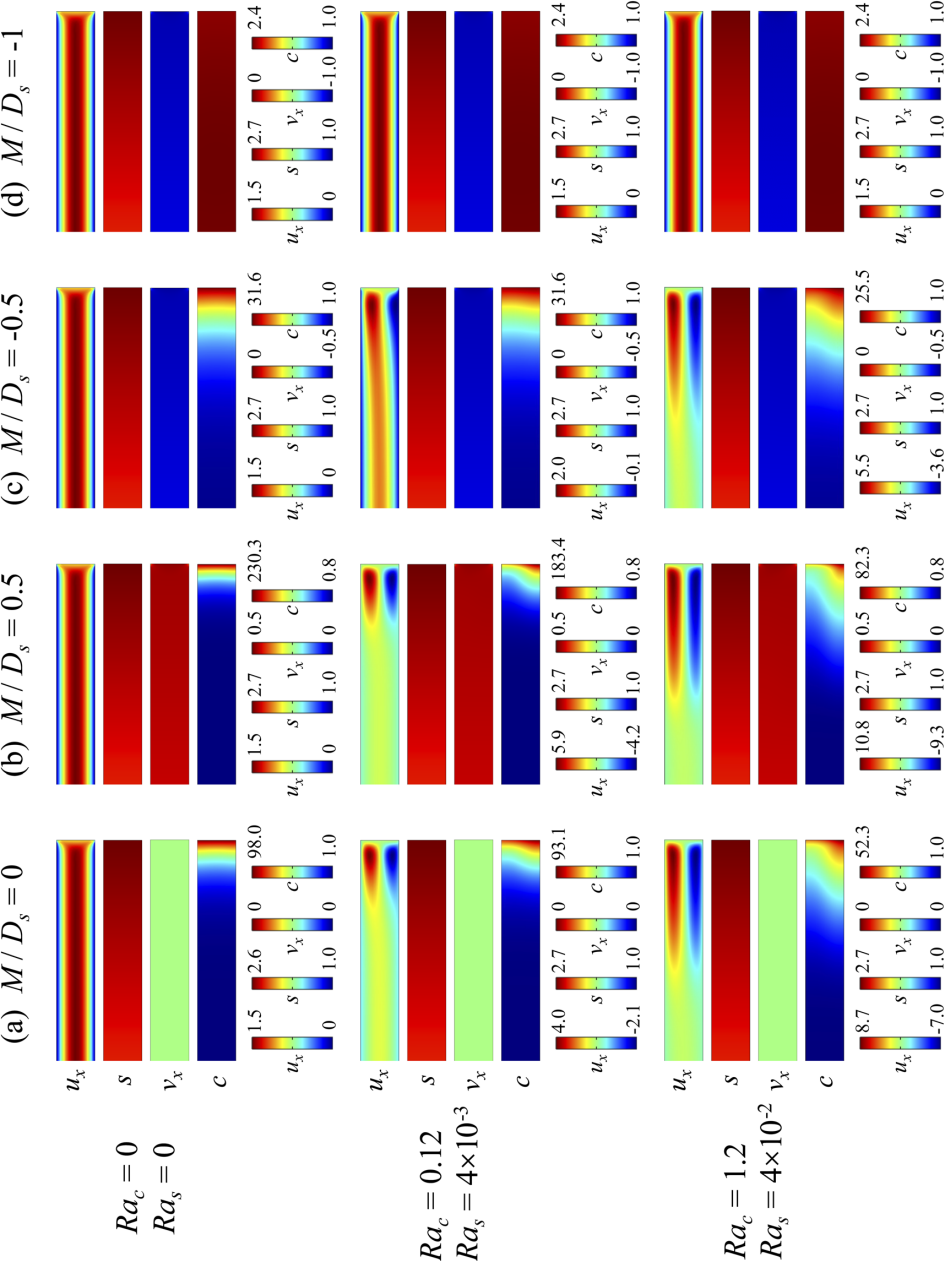
Density profiles of the *x*-component fluid velocity, *u*_x_, solute concentration, *s*, *x*-component diffusiophoretic velocity, *v*_x_, and colloid concentration, *c*, at *t* = 10^4^ for different Ra_c_ and Ra_s_ with *D*_c_/*D*_s_ = 10^−2^ and Pe = 10^−2^. The density profiles are obtained with channel length *l* = 10^3^ but only the sections next to the drying interface, *x* ∈ [−10, 0], are shown to illustrate important physics. (a) *M*/*D*_s_ = 0; no diffusiophoresis. (b) *M*/*D*_s_ = 0.5; solute-attracted diffusiophoresis. (c) *M*/*D*_s_ = −0.5; weakly solute-repelled diffusiophoresis. (d) *M*/*D*_s_ = −1; strongly solute-repelled diffusiophoresis.

Fig. 5 shows twelve sets of density profiles of the *x*-component solvent velocity, *u*_x_, solute concentration, *s*, *x*-component diffusiophoretic velocity, *v*_x_, and colloid concentration, *c*, at *t* = 10^4^ for different Ra_c_, Ra_s_, and *M*/*D*_*s*_, with *D*_c_/*D*_s_ = 10^−2^ and Pe = 10^−2^. In practice, varying Ra_c_ and Ra_s_ can be achieved by matching the density of the colloids and the solvent,^68,69^ changing the initial colloid and solute concentration, or conducting unidirectional drying under microgravity. Similar to Fig. 2, the top and bottom of each density plot corresponds to the channel walls at *z* = 1 and *z* = 0, respectively. The right-end of each density plot corresponds to the drying interface at *x* = 0. Only the section of the channel next to the drying interface, *x* ∈ [−10, 0], is shown to illustrate important physics. The vertical bars below each set of density profiles show the values of the density profiles.

Let us first examine Fig. 5(a) which is obtained with no diffusiophoresis, *M*/*D*_s_ = 0. In the absence of gravitational effect, Ra_c_ = Ra_s_ = 0, colloid (∂*c*/∂*x*) and solute (∂*s*/∂*x*) concentration gradients do not cause solvent backflow according to eqn (20). Thus, the solvent flow profile, *u*_x_, remains parabolic, with the maximum velocity along the channel centerline and zero velocity at the channel walls. As Ra_c_ and Ra_s_ become non-zero, gravity causes sedimentation of colloids and induces a backflow of the suspension, indicated by a negative *u*_x_. As Ra_c_ and Ra_s_ continue to increase in the third set of density profiles in Fig. 5(a), the maximum *u*_x_ and *c*, which occur near the drying interface, increases and decreases, respectively. This can be understood as follows. According to eqn (20), the magnitude of the backflow *u*_x,back_ increases as Ra_c_ and Ra_s_ increase. A larger backflow carries more colloids to the left and hence decreases the maximum *c* near the drying interface. Note that, the backflow also weakens the colloid and solute concentration gradients which in turn has a weakening effect on the solvent backflow. However, the weakening of the backflow induced by decreasing the colloid concentration gradient is smaller than the strengthening of the backflow due to increasing Ra_c_ and Ra_s_. As a result, from eqn (20), overall the backflow velocity increases as Ra_c_ and Ra_s_ increase.

Next, let us look at Fig. 5(b) which is obtained with solute-attracted diffusiophoresis, *M*/*D*_s_ = 0.5. In the absence of gravitational effect, Ra_c_ = Ra_s_ = 0, comparing Fig. 5(b) and (a), the solvent velocity *u*_x_ in panel (b) and (a) are identical whereas the colloid concentration *c* and concentration gradient ∂*c*/∂*x* in (b) are higher than those in (a). In the presence of gravity, Ra_c_ = [0.12, 1.2] and Ra_s_ = [4 × 10^−3^, 4 × 10^−2^], the magnitude of *u*_x_ and *c* in panel (b) are higher than those in panel (a) at the same time *t*, although the profiles of *u*_x_, *s*, and *c* between panel (b) and (a) at the same *t* are qualitatively the same. Here, the physical explanations are the same as those in comparing Fig. 2(b) and (a) and we do not repeat them.

Next, let us examine Fig. 5(c) which is obtained with weakly solute-repelled diffusiophoresis, *M*/*D*_s_ = −0.5. When Ra_c_ = Ra_s_ = 0, comparing Fig. 5(c) and (a), *u*_x_ in panel (c) and (a) are identical whereas *c* and d*c*/d*x* in (c) are lower than those in (a). When Ra_c_ = [0.12, 1.2] and Ra_s_ = [4 × 10^−3^, 4 × 10^−2^], the magnitude of *u*_x_ and *c* in panel (c) are lower than those in panel (a) at the same time *t*, although the profiles of *u*_x_, *s*, and *c* between panel (b) and (a) at the same *t* are qualitatively the same. Again, the physical explanations here are identical to those in comparing Fig. 2(c) and (a) and we do not repeat them.

Next, let us look at Fig. 5(d) which is obtained with strongly solute-repelled diffusiophoresis, *M*/*D*_s_ = −1. Notably, all density profiles are almost invariant under different Ra_c_ and Ra_s_. We understand this by recalling Fig. 2(d) that strongly solute-repelled diffusiophoresis prevents the formation of a strong colloid concentration gradient, ∂*c*/∂*x* → 0. Thus, according to eqn (20), solvent backflow is absent. It follows that the evaporation-induced parabolic flow profile persists, regardless of the value of Ra_c_ and Ra_s_. On a different note, comparing to Fig. 5(a)–(c), strongly solute-repelled diffusiophoresis in Fig. 5(d) leads to the weakest phase separation and develops the thickest colloidal layer. The colloidal layer formed in Fig. 5(d) is also the most uniform across the *z*-direction among all cases. In the next section, we quantify the time evolution of the colloidal layer thickness and mean position of the colloid distribution in the above cases.

#### 3.2.2 Colloidal layer thickness and mean colloid position

**Fig. 6:**
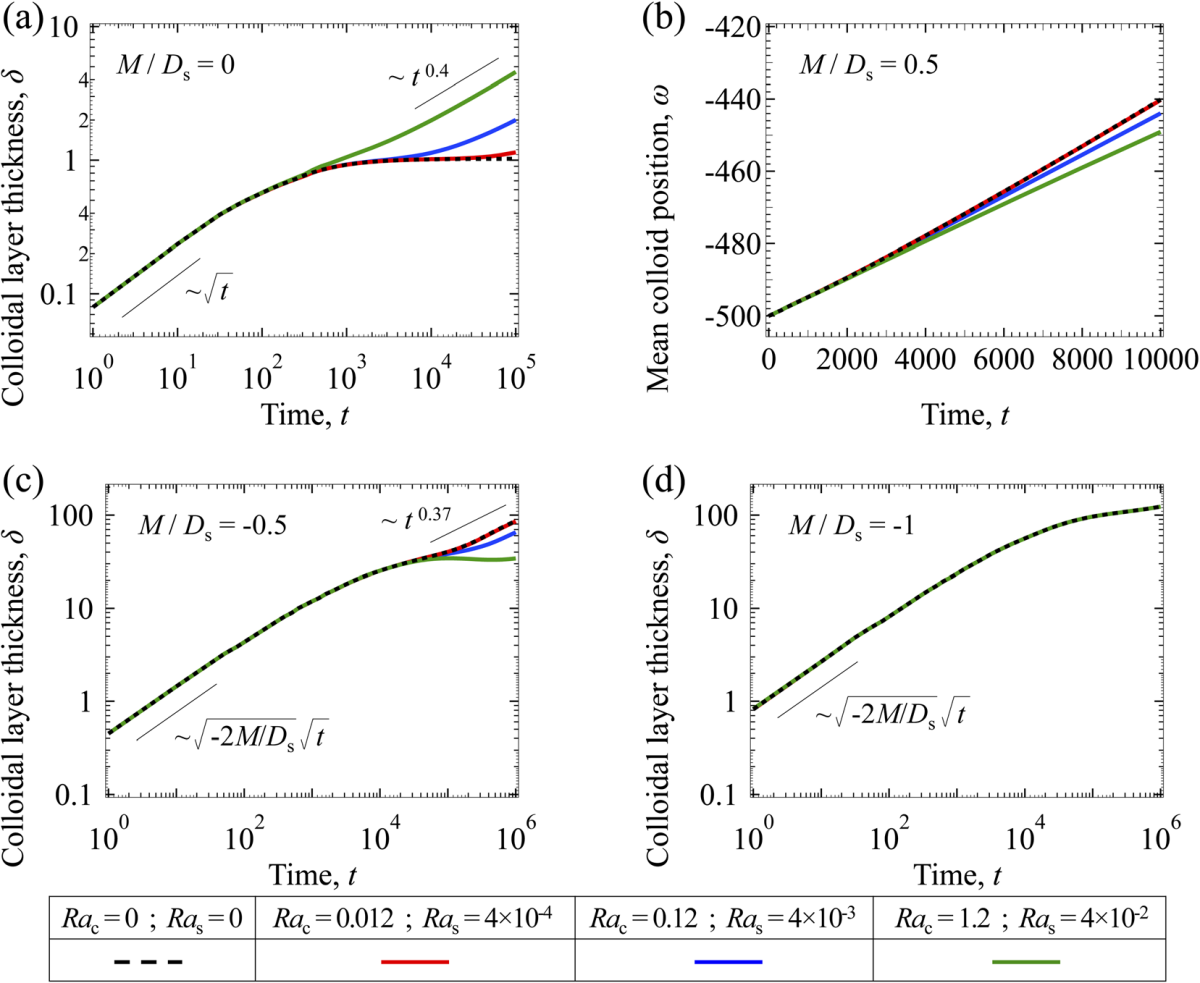
Time evolution of the (a, c and d) colloidal layer thickness, *δ*, and (b) mean position of the colloid distribution, *ω*, for different Ra_c_ and Ra_s_ with *D*_c_/*D*_s_ = 10^−2^ and Pe = 10^−2^. (a) *M*/*D*_s_ = 0; no diffusiophoresis. (b) *M*/*D*_s_ = 0.5; solute-attracted diffusiophoresis. (c) *M*/*D*_s_ = −0.5; weakly solute-repelled diffusiophoresis. (d) *M*/*D*_s_ = −1; strongly solute-repelled diffusiophoresis.

Let us first examine the case with no diffusiophoresis. Fig. 6(a) shows the time evolution of the colloidal layer thickness, *δ*, for different Ra_c_ and Ra_s_, with *M*/*D*_s_ = 0, *D*_c_/*D*_s_ = 10^−2^, and Pe = 10^−2^. Here, the green line with strong gravitational effects, Ra_c_ = 1.2 and Ra_s_ = 4 × 10^−2^, is identical to the dashed line in Fig. 3(a), where *δ* grows as 
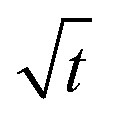
 at early times and *t*^0.4^ at long times. As gravitational effects weaken, represented by a decrease in Ra_c_ and Ra_s_, the early-time diffusive scaling persists. However, the long-time scaling weakens and eventually reaches a plateau in the limit of Ra_c_ = Ra_s_ = 0 [eqn (38)]. In sum, in the absence of diffusiophoresis, while gravity has negligible effects on electrolyte-colloid phase separation and thus the colloidal layer thickness at early times, an increasing gravitational effect weakens phase separation and develops a thicker colloidal layer at long times. These results recover the key findings in prior work.^21^

Next, let us analyze the case with solute-attracted diffusiophoresis in Fig. 6(b). As noted in Section 3.1.2, the traditional definition of *δ* is not applicable to quantify the transport of solute-attracted diffusiophoretic colloids but the mean position of the colloid distribution, *ω*, could be measured instead. Fig. 6(b) shows the time evolution of *ω* for different Ra_c_ and Ra_s_, with *M*/*D*_s_ = 0.5, *D*_c_/*D*_s_ = 10^−2^, and Pe = 10^−2^. Fig. 6(b) shows that *ω* becomes more negative as Ra_c_ and Ra_s_ increase. Physically, larger Ra_c_ and Ra_s_ imply a larger backflow [eqn (20)], which transports more colloids away from the drying interface. This shifts the mean position of the colloid concentration distribution to the left and therefore *ω* becomes more negative. In other words, similar to the case with no diffusiophoresis, in the presence of solute-attracted diffusiophoresis, gravity has negligible effects on electrolyte-colloid phase separation and thus the colloidal layer thickness at early times. However, an increasing gravitational effect leads to a stronger solvent backflow, which weakens phase separation and develops a thicker colloidal layer at long times.

Next, let us analyze the case with weakly solute-repelled diffusiophoresis, *M*/*D*_s_ = −0.5, in Fig. 6(c). Here, the green line with strong gravitational effects, Ra_c_ = 1.2 and Ra_s_ = 4 × 10^−2^, is identical to the red line in Fig. 3(a), where *δ* grows as 
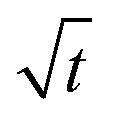
 at early times and plateaus at long times. As gravitational effects weaken, the early-time diffusive scaling persists. However, the long-time scaling grows and eventually becomes *t*^0.37^ in the limit of Ra_c_ = Ra_s_ = 0. We note that the origin of the *t*^0.37^ scaling is different from the similar *t*^0.4^ scaling in Fig. 6(a). Specifically, the *t*^0.37^ scaling of the dashed line in Fig. 6(c) is associated with a system with diffusiophoresis but no gravitational effects, whereas the *t*^0.4^ scaling of the dashed line in Fig. 6(c) is associated with a system under gravity but without diffusiophoresis. Our simulations show that the *t*^0.37^ scaling is still evolving at *t* = 10^6^ but the *t*^0.4^ scaling is reached and invariant at *t* ≥ 10^4^. On a different note, due to the competition between colloid transport induced by gravity and diffusiophoresis, increasing gravity strengthens phase separation and decreases *δ* in the presence of diffusiophoresis [Fig. 6(c)] whereas increasing gravity weakens phase separation and increases *δ* in the absence of diffusiophoresis [Fig. 6(a)]. This demonstrates another qualitative impact of diffusiophoresis on unidirectional drying, in addition to the order-of-magnitude enhancement in *δ* exhibited in Fig. 3(a).

Lastly, we show the time evolution of *δ* with strongly solute-repelled diffusiophoresis, *M*/*D*_s_ = −1, in Fig. 6(d). The green line with strong gravitational effects, Ra_c_ = 1.2 and Ra_s_ = 4 × 10^−2^, is identical to the green line in Fig. 3(a). Here, the data for different Ra_c_ and Ra_s_ overlaps onto the same line, meaning that under strongly solute-repelled diffusiophoresis gravity has no effect on phase separation and therefore *δ*. This is due to the absence of solvent backflow as explained in Fig. 5(d). As an overview of Fig. 6(a)–(d), the effect of gravity on *δ* is the most prominent when diffusiophoresis is absent [Fig. 6(a)]. Diffusiophoresis could delay [Fig. 6(b) and (c)] or even eliminate [Fig. 6(d)] the impact of gravity on *δ*.

## 4 Conclusions

In this work, we have utilized direct numerical simulations and developed a macrotransport theory to quantify the advective–diffusive transport of diffusiophoretic colloids in a unidirectional drying cell. We focus on analyzing the time evolution of the solvent velocity *u*_x_, solute concentration field *s*, colloid diffusiophoretic velocity *v*_x_, colloid concentration field *c*, colloidal layer thickness *δ*, and mean position of the colloid distribution *ω*.

The first part of our analyses focuses on the impact of varying diffusiophoresis under constant, non-zero gravity. At long times, as the colloids switch from solute-attracted to solute-repelled (*M*/*D*_s_ becomes more negative), the magnitude of *u*_x_ and *c* near the drying interface decreases. Solvent backflow is absent in a suspension of strongly solute-repelled colloids, since diffusiophoresis prevents the formation of a strong colloid concentration gradient. We further quantify *δ*. The scalings of *δ* obtained from our macrotransport theory agree with simulations. For weakly solute-repelled colloids, *δ* grows diffusively at early times and plateaus at long times. For strongly solute-repelled colloids, *δ* also grows diffusively initially but continues to grow at long times. The colloidal layer thickness of solute-attracted colloids cannot be quantified by the traditional formula for non-diffusiophoretic colloids. Thus, we compute the mean position of the colloid distribution and show that diffusiophoresis concentrates solute-attracted colloids near the drying interface where the solute accumulates. Overall, phase separation is the strongest and weakest with solute-attracted and solute-repelled colloids, respectively. The colloidal layer formed by solute-repelled colloids could be ten times thicker than that by non-diffusiophoretic colloids.

The second part of our analyses focuses on the impact of varying gravity at long times. In the absence of gravity, *u*_x_ is independent of the strength of diffusiophoresis and the colloid concentration near the drying interface decreases as the colloids switch from solute-attracted to solute-repelled. In the presence of constant non-zero gravity, as *M*/*D*_s_ becomes more negative, the magnitude of *u*_x_ and *c* near the drying interface decreases. A suspension of strongly solute-repelled colloids is a special case. Strongly solute-repelled diffusiophoresis prevents the formation of a significant colloid concentration gradient and the subsequent solvent backflow. As a result, all four quantities *u*_x_, *s*, *v*_x_, and *c* are invariant regardless of the strength of gravity. We further quantify *δ* and *ω*. Increasing gravity is shown to weaken phase separation and increase *δ* in the absence of diffusiophoresis whereas gravity has the opposite effect on phase separation and *δ* in the presence of weakly solute-repelled diffusiophoresis. For strongly solute-repelled colloids, gravity has no effect on phase separation and *δ*.

The present work considers dilute colloidal suspensions and highlights the important role of diffusiophoresis in colloidal film formation. For future work, the present model could be extended to consider channel walls with non-uniform electrokinetic properties^70–73^ as well as converging or diverging channels.^44^ We expect that these factors will have qualitative impacts on the thickness and uniformity of the colloidal layer. To quantify the formation of a dense colloidal film, one could turn to particle dynamics simulations that account for the finite size of particles. Some recent work has been done in this direction^74–79^ but, to the authors' knowledge, the electrophoretic component of diffusiophoresis has been ignored. It will be of interest to conduct particle dynamics simulations that include complete diffusiophoresis and compare with present results for the development of a reduced-order model.

## Appendix A: a macrotransport theory for diffusiophoretic colloids under gravity

In this section, we derive a macrotransport theory that predicts the growth of the colloidal layer thickness, *δ*, presented in Sections 3.1.2 and 3.2.2. In the following, we start with deriving the theory for an electrolyte-colloid suspension under gravity with diffusiophoresis, which is a novel result of this work. Then, we will show that our theory could reduce to that for a non-electrolyte-colloid suspension under gravity with no diffusiophoresis developed in prior work.^21^

To derive the macrotransport theory for an electrolyte-colloid suspension under gravity with diffusiophoresis, first we recall the two-dimensional colloid transport eqn (19)21
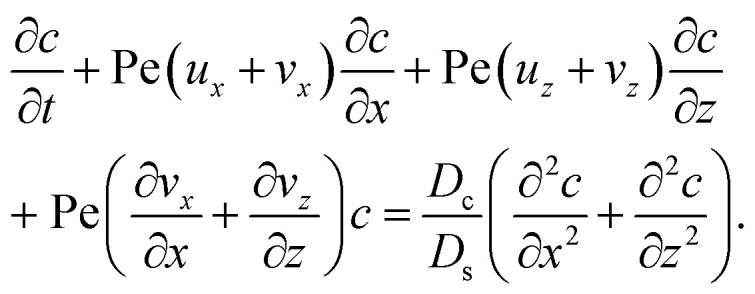
Following prior work in macrotransport theory,^80,81^ the colloid concentration field, *c*, is written as in terms of its cross-sectional average, *c*_0_ = 〈*c*〉, and variation from the average, Pe*c*_1_,

22 *c*(*x*,*z*,*t*) = *c*_0_(*x*,*t*) + Pe*c*_1_(*x*,*z*,*t*),

where Pe*c*_1_ ≪ *c*_0_ and the cross-sectional average is 
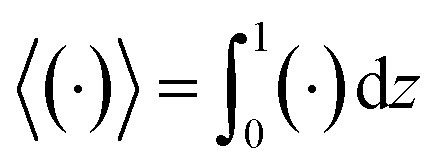
. Substituting eqn (22) into (21) and performing cross-sectional averaging gives23
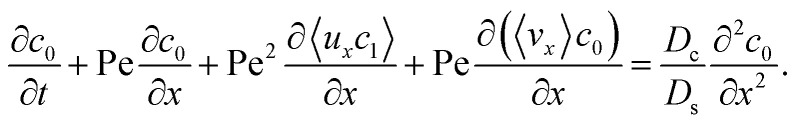
The objective now is to obtain *u*_x_ and *c*_1_ and then substitute them into eqn (23).

To obtain *u*_x_, we invoke the continuity equation that gives *u*_z_ ∼ *u*_x_/*δ* and assume *δ* ≫ 1 so that *u*_x_ ≫ *u*_z_ ≈ 0 and ∂*u*_x_/∂*x* ≈ 0. Using these conditions in eqn (15) and (16) gives24
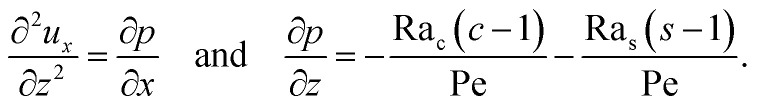
The flow field *u*_x_ comprises a pressure-driven flow induced by solvent evaporation, *u*^p^_x_, and a flow induced by gravity, *u*^g^_x_,^82^*i.e.*, *u*_x_ = *u*^p^_x_ + *u*^g^_x_. By linearity of the equations, *u*^p^_x_ and *u*^g^_x_ can be obtained as25
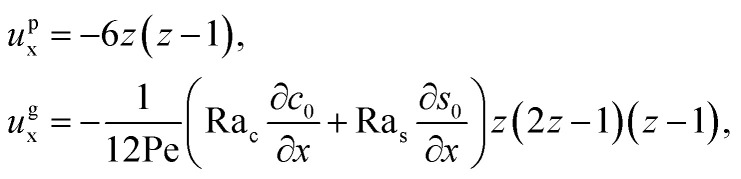
where both expressions satisfy the no-slip condition at the channel walls, *u*_x_ = 0 at *z* = 0 and *z* = 1, and mass conservation 〈*u*^p^_x_〉 = 1 and 〈*u*^g^_x_〉 = 0.

Next, to obtain *c*_1_, we subtract eqn (23) from eqn (21), along with the use of eqn (22), to give26
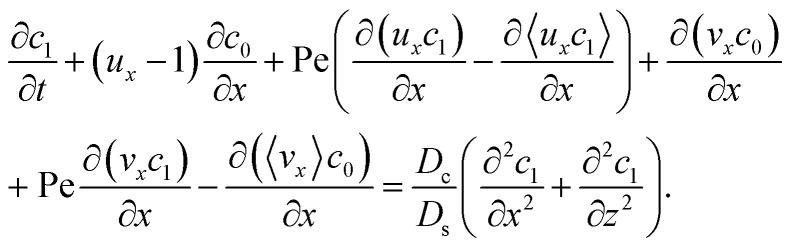
Considering *t* ≫ *D*_s_/*D*_c_ where diffusion has made the colloid distribution largely uniform along the *z*-direction, eqn (26) reduces to27
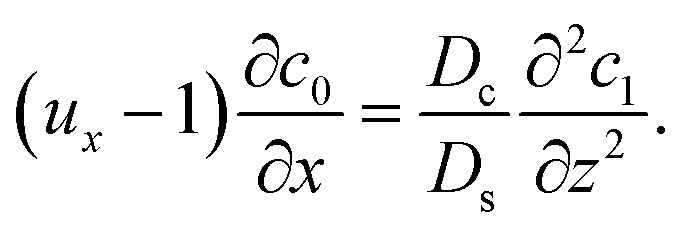
Similar to the flow field, *c*_1_ can be written as a sum of a component due to the pressure-driven flow induced by solvent evaporation, *c*^p^_1_, and a component due to the flow induced by gravity, *c*^g^_1_. By linearity of the equations, *c*^p^_1_ and *c*^g^_1_ can be obtained as28
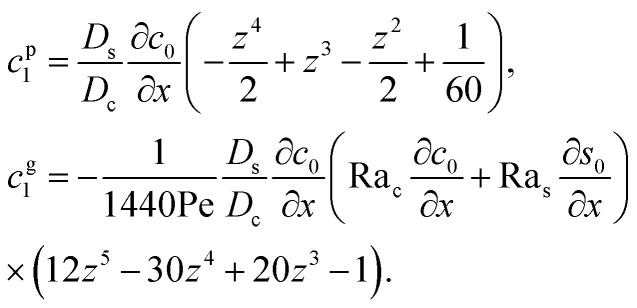


Substituting eqn (25) and (28) in (23) gives the macrotransport equation for an electrolyte-colloid suspension under gravity with diffusiophoresis29
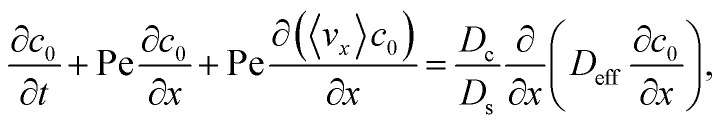
with30
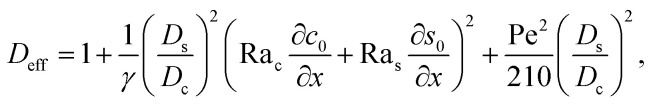
where *γ* = 362 880. In eqn (30), the first term is due to intrinsic colloid diffusion, the second term is due to gravity-induced dispersion, and the third term is due to evaporation-induced dispersion. In the second term, Ra_s_(∂*s*_0_/∂*x*) is negligible relative to Ra_c_(∂*c*_0_/∂*x*). Further, the third term is negligible compared to the first two terms. These two simplifications are supported by the typical physical parameters in Section 2 and time evolution of *c*_0_ and *s*_0_ in Section 3. With these simplifications, eqn (29) can be arranged as31
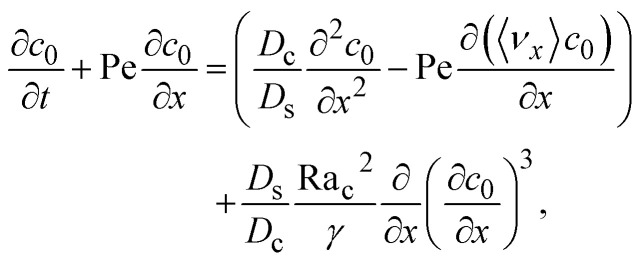
where the order of magnitudes of these four positive terms are32
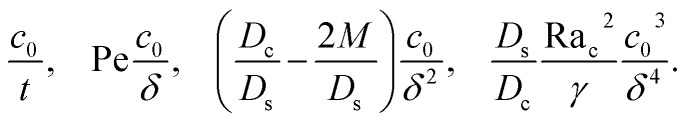
We restrict *M* to be non-positive here to give scalings of a positive and physically relevant *δ*. Several scalings of *δ* can be obtained. First, at early times, regardless of the strength of gravity and when diffusiophoresis is present, a balance between the transient *c*_0_/*t* and diffusiophoretic transport (*D*_c_/*D*_s_ − 2*M*/*D*_s_)*c*_0_/*δ*^2^ gives33
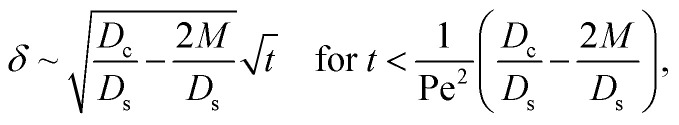
where the range of *t* is obtained by the inequality *c*_0_/*t* > Pe*c*_0_/*δ*. Second, at long times when there is weakly solute-repelled diffusiophoresis under strong gravity, a balance between convection Pe*c*_0_/*δ* and diffusiophoresis (*D*_c_/*D*_s_ − 2*M*/*D*_s_)*c*_0_/*δ*^2^ gives34
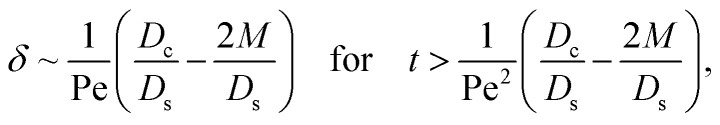
where the range of *t* is obtained by the inequality *c*_0_/*t* < Pe*c*_0_/*δ*. This completes the macrotransport theory and the scalings of *δ* for an electrolyte-colloid suspension under gravity with diffusiophoresis.

Next, we show that the above theory could reduce to that for a non-electrolyte-colloid suspension under gravity with no diffusiophoresis developed in prior work.^21^ In the absence of electrolytes and hence diffusiophoresis, *v*_x_ = 0 and eqn (31) reduces to35
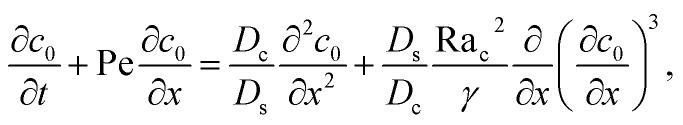
where the order of magnitudes of these four positive terms are36
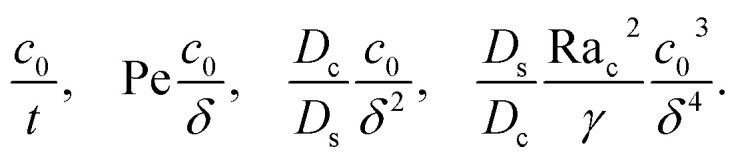
Note that, in the absence of electrolytes, all quantities associated with the solute concentration, *e.g.*, Ra_s_, *s*, and *s*_0_, vanish, except *D*_s_. Here, *D*_s_ does not bear any physical meaning and is merely a reference diffusivity that constitutes the same non-dimensionalization scheme used in the macrotransport theory for an electrolyte-colloid suspension. Several scalings of *δ* can be obtained. First, at early times, regardless of the strength of gravity, a balance between the transient *c*_0_/*t* and diffusion term *D*_c_*c*_0_/(*D*_s_*δ*^2^) gives,37
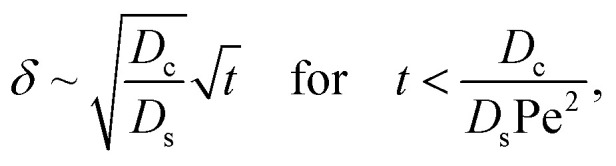
where the range of *t* is obtained by the inequality *c*_0_/*t* > Pe*c*_0_/*δ*. Second, at long times when gravity is absent, a balance between convection Pe*c*_0_/*δ* and diffusion *D*_c_*c*_0_/(*D*_s_*δ*^2^) gives38
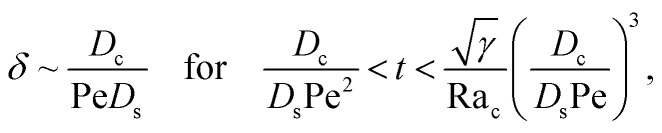
where mass conservation 
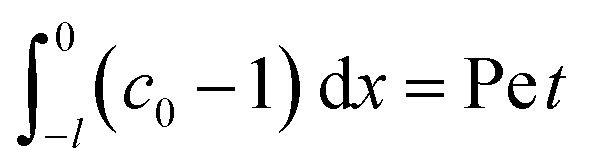
 and its scaling *c*_0_*δ* ∼ Pe*t* have been invoked. The range of *t* is obtained by the inequalities *c*_0_/*t* < Pe*c*_0_/*δ* and *D*_c_*c*_0_/(*D*_s_*δ*^2^) > *D*_s_Ra_c_^2^*c*_0_^3^/(*D*_c_*γδ*^4^). Third, at long times when gravity is present a balance between convection Pe*c*_0_/*δ* and gravity *D*_s_Ra_c_^2^*c*_0_^3^/(*D*_c_*γδ*^4^) gives39
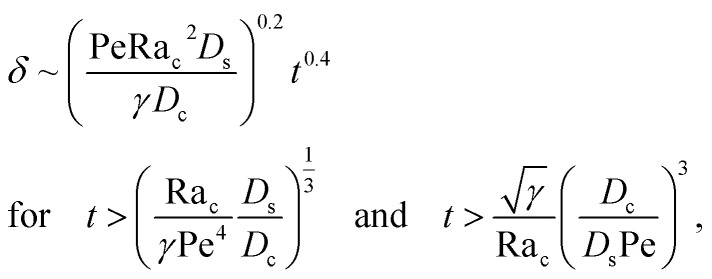
where the range of *t* is obtained by the inequalities *c*_0_/*t* < Pe*c*_0_/*δ* and *D*_c_*c*_0_/(*D*_s_*δ*^2^)<*D*_s_Ra_c_^2^*c*_0_^3^/(*D*_c_*γδ*^4^). We have checked that the above scalings of *δ* and the corresponding range of *t* agree with results obtained from direct numerical simulations in Section 3, confirming the validity of the simulations and the macrotransport theory.

## Conflicts of interest

There are no conflicts of interest to declare.

## Supplementary Material

## References

[cit1] Luo H., Cardinal C. M., Scriven L. E., Francis L. F. (2008). Langmuir.

[cit2] Inasawa S., Yamaguchi Y. (2009). Langmuir.

[cit3] Yamaguchi K., Inasawa S., Yamaguchi Y. (2013). Phys. Chem. Chem. Phys..

[cit4] Utgenannt A., Maspero R., Fortini A., Turner R., Florescu M., Jeynes C., Kanaras A. G., Muskens O. L., Sear R. P., Keddie J. L. (2016). ACS Nano.

[cit5] Stein IV M., Mistry A., Mukherjee P. P. (2017). J. Electrochem. Soc..

[cit6] Bacchin P., Brutin D., Davaille A., Giuseppe E. D., Chen X. D., Gergianakis I., Giorgiutti-Dauphiné F., Goehring L., Hallez Y., Heyd R., Jeantet R., Floch-Fouéré C. L., Meireles M., Mittelstaedt E., Nicloux C., Pauchard L., Saboungi M.-L. (2018). Eur. Phys. J. E: Soft Matter Biol. Phys..

[cit7] Tinkler J. D., Scacchi A., Kothari H. R., Tulliver H., Argaiz M., Archer A. J., Martín-Fabiani I. (2021). J. Colloid Interface Sci..

[cit8] Allain C., Limat L. (1995). Phys. Rev. Lett..

[cit9] Dufresne E. R., Corwin E. I., Greenblatt N. A., Ashmore J., Wang D. Y., Dinsmore A. D., Cheng J. X., Xie X. S., Hutchinson J. W., Weitz D. A. (2003). Phys. Rev. Lett..

[cit10] Moreau P., Dehmoune J., Salmon J.-B., Leng J. (2009). Appl. Phys. Lett..

[cit11] Sarkar A., Tirumkudulu M. S. (2009). Langmuir.

[cit12] Gauthier G., Lazarus V., Pauchard L. (2010). Europhys. Lett..

[cit13] Inasawa S., Yamaguchi Y. (2012). Soft Matter.

[cit14] Routh A. F. (2013). Rep. Prog. Phys..

[cit15] Daubersies L., Leng J., Salmon J.-B. (2013). Lab Chip.

[cit16] Lidon P., Salmon J.-B. (2014). Soft Matter.

[cit17] Goehring L., Li J., Kiatkirakajorn P.-C. (2017). Philos. Trans. R. Soc., A.

[cit18] Abe K., Inasawa S. (2018). Phys. Chem. Chem. Phys..

[cit19] Inoue K., Inasawa S. (2020). RSC Adv..

[cit20] Lee D., Kim J., Lee H., Kim S. J. (2020). Micro Nano Syst. Lett..

[cit21] Salmon J.-B., Doumenc F. (2020). Phys. Rev. Fluids.

[cit22] Prieve D. C., Roman R. (1987). J. Chem. Soc., Faraday Trans. 2.

[cit23] Anderson J. L. (1989). Annu. Rev. Fluid. Mech..

[cit24] Keh H. J. (2016). Curr. Opin. Colloid Interface Sci..

[cit25] Velegol D., Garg A., Guha R., Kar A., Kumar M. (2016). Soft Matter.

[cit26] Marbach S., Bocquet L. (2019). Chem. Soc. Rev..

[cit27] Abecassis B., Cottin-Bizonne C., Ybert C., Ajdari A., Bocquet L. (2009). New J. Phys..

[cit28] Palacci J., Abecassis B., Cottin-Bizonne C., Ybert C., Bocquet L. (2010). Phys. Rev. Lett..

[cit29] Deseigne J., Cottin-Bizonne C., Stroock A. D., Bocquet L., Ybert C. (2014). Soft Matter.

[cit30] Volk R., Mauger C., Bourgoin M., Cottin-Bizonne C., Ybert C., Raynal F. (2014). Phys. Rev. E: Stat., Nonlinear, Soft Matter Phys..

[cit31] Banerjee A., Williams I., Azevedo R. N., Helgeson M. E., Squires T. M. (2016). Proc. Natl. Acad. Sci. U. S. A..

[cit32] Mauger C., Volk R., Machicoane N., Bourgoin M., Cottin-Bizonne C., Ybert C., Raynal F. (2016). Phys. Rev. Fluids.

[cit33] Shi N., Nery-Azevedo R., Abdel-Fattah A. I., Squires T. M. (2016). Phys. Rev. Lett..

[cit34] Shin S., Um E., Sabass B., Ault J. T., Rahimi M., Warren P. B., Stone H. A. (2016). Proc. Natl. Acad. Sci. U. S. A..

[cit35] Ault J. T., Warren P. B., Shin S., Stone H. A. (2017). Soft Matter.

[cit36] Friedrich S. M., Burke J. M., Liu K. J., Ivory C. F., Wang T. (2017). Nat. Commun..

[cit37] Shukla V., Volk R., Bourgoin M., Pumir A. (2017). New J. Phys..

[cit38] Shin S., Ault J. T., Warren P. B., Stone H. A. (2017). Phys. Rev. X.

[cit39] Ault J. T., Shin S., Stone H. A. (2018). J. Fluid Mech..

[cit40] Raynal F., Bourgoin M., Cottin-Bizonne C., Ybert C., Volk R. (2018). J. Fluid Mech..

[cit41] Chu H. C. W., Garoff S., Tilton R. D., Khair A. S. (2021). J. Fluid Mech..

[cit42] Chu H. C. W., Garoff S., Tilton R. D., Khair A. S. (2020). Soft Matter.

[cit43] Shimokusu T. J., Maybruck V. G., Ault J. T., Shin S. (2020). Langmuir.

[cit44] Rasmussen M. K., Pedersen J. N., Marie R. (2020). Nat. Commun..

[cit45] Chu H. C. W., Garoff S., Tilton R. D., Khair A. S. (2022). Soft Matter.

[cit46] Migacz R. E., Ault J. T. (2022). Phys. Rev. Fluids.

[cit47] Volk R., Bourgoin M., Brehier C.-E., Raynal F. (2022). J. Fluid Mech..

[cit48] McKenzie B. E., Chu H. C. W., Garoff S., Tilton R. D., Khair A. S. (2022). J. Fluid Mech..

[cit49] Sambamoorthy S., Chu H. C. W. (2023). Soft Matter.

[cit50] Shi N., Abdel-Fattah A. (2021). Phys. Rev. Fluids.

[cit51] Tan H., Banejee A., Shi N., Tang X., Abdel-Fattah A., Squires T. M. (2021). Sci. Adv..

[cit52] Park S. W., Lee J., Yoon H., Shin S. (2021). Energy Fuels.

[cit53] Shin S., Doan V. S., Feng J. (2019). Phys. Rev. Appl..

[cit54] Doan V. S., Chun S., Feng J., Shin S. (2021). Nano Lett..

[cit55] Sear R. P., Warren P. B. (2017). Phys. Rev. E.

[cit56] Deen W. M. , Analysis of Transport Phenomena, Oxford University Press, New York, 2012

[cit57] Gu Y., Hegde V., Bishop K. J. M. (2018). Lab Chip.

[cit58] Shklyaev O. E., Yashin V. V., Stupp S. I., Balazs A. C. (2020). Commun. Phys..

[cit59] Williams I., Lee S., Apriceno A., Sear R. P., Battaglia G. (2020). Proc. Natl. Acad. Sci. U. S. A..

[cit60] Hjerten S. (1985). J. Chromatogr. A.

[cit61] Kaniansky D., Masár M., Bielcíková J. (1997). J. Chromatogr. A.

[cit62] Gupta A., Shim S., Stone H. A. (2020). Soft Matter.

[cit63] Romankiw L. A., Chou I.-M. (1983). J. Chem. Eng. Data.

[cit64] Pitzer K. S., Peiper J. C., Busey R. H. (1984). J. Phys. Chem. Ref. Data.

[cit65] Gates J. A., Wood R. H. (1985). J. Chem. Eng. Data.

[cit66] Pradhan T. K., Panigrahi P. K. (2016). Microfluid. Nanofluid..

[cit67] Squires T. M., Quake S. R. (2005). Rev. Mod. Phys..

[cit68] Wiederseiner S., Andreini N., Epely-Chauvin G., Ancey C. (2011). Exp. Fluids.

[cit69] Poon W. C. K., Weeks E. R., Royall C. P. (2012). Soft Matter.

[cit70] Khair A. S., Squires T. M. (2008). Phys. Fluids.

[cit71] Ng C.-O., Chu H. C. W. (2011). Phys. Fluids.

[cit72] Chow K. W., Chu H. C. W., Ng C.-O. (2013). Int. J. Nonlinear Sci. Numer. Simul..

[cit73] Michelin S., Game S., Lauga E., Keaveny E., Papageorgiou D. (2020). Soft Matter.

[cit74] Fortini A., Sear R. P. (2017). Langmuir.

[cit75] Schulz M., Keddie J. L. (2018). Soft Matter.

[cit76] Statt A., Howard M. P., Panagiotopoulos A. Z. (2018). J. Chem. Phys..

[cit77] Tatsumi R., Iwao T., Koike O., Yamaguchi Y., Tsuji Y. (2018). Appl. Phys. Lett..

[cit78] Tang Y., Grest G. S., Cheng S. (2019). Langmuir.

[cit79] Jeong J. H., Lee Y. K., Ahn K. H. (2021). AIChE J..

[cit80] Taylor G. (1953). Proc. R. Soc. London, Ser. A.

[cit81] Brenner H. and Edwards D. A., Macrotransport Processes, Butterworth-Heinemann, Massachusetts, 1993

[cit82] Birikh R. V. (1966). J. Appl. Mech. Tech. Phys..

